# Lapping and Polishing of Crystalline KY(WO_4_)_2_: Toward High Refractive Index Contrast Slab Waveguides

**DOI:** 10.3390/mi10100674

**Published:** 2019-10-04

**Authors:** Carlijn I. van Emmerik, Roy Kooijman, Meindert Dijkstra, Sonia M. Garcia-Blanco

**Affiliations:** 1 Optical Sciences, MESA+ Institute for Nanotechnology, University of Twente, 7500 AE Enschede, The Netherlands; m.dijkstra@utwente.nl (M.D.);; 2 Techno Centrum voor Onderwijs en Onderzoek, Glas instrumentmakerij, University of Twente, Drienerlolaan 5, 7522 NB Enschede, The Netherlands; r.kooijman@phix.com; 3 Currently at PHIX, De Veldmaat 17, 7522 NM Enschede, The Netherlands

**Keywords:** integrated optics, heterogeneous integration, lapping, polishing

## Abstract

Rare-earth ion-doped potassium yttrium double tungstate, RE:KY(WO_4_)_2_, is a promising candidate for small, power-efficient, on-chip lasers and amplifiers. Thin KY(WO_4_)_2_-on-glass layers with thicknesses ranging between 0.9 and 1.6 μm are required to realize on-chip lasers based on high refractive index contrast waveguides operating between 1.55 and 3.00 µm. The crystalline nature of KY(WO_4_)_2_ makes the growth of thin, defect-free layers on amorphous glass substrates impossible. Heterogeneous integration is one of the promising approaches to achieve thin KY(WO_4_)_2_-on-glass layers. In this process, crystal samples, with a thickness of 1 mm, are bonded onto a glass substrate and thinned down with an extensive lapping and polishing procedure to the desired final thickness. In this study, a lapping and polishing process for KY(WO_4_)_2_ was developed toward the realization of integrated active optical devices in this material.

## 1. Introduction

Potassium double tungstate (i.e., KY(WO_4_)_2_, KGd(WO_4_)_2_, and KLu(WO_4_)_2_) was used for decades as an active material for Raman lasers [1,2,3] and, when doped with rare-earth ions, for high-power ultra-short pulsed lasers [4,5] and thin disk lasers [6,7]. The large interionic distance (⟨d⟩≈0.4 nm [8]) of KY(WO_4_)_2_ allows it to be doped with high concentrations of rare-earth ions [9,10,11], without experiencing quenching effects. The crystal provides high emission and absorption cross-section for rare-earth ions doped in it [11]. These properties, in combination with its relatively high refractive index (n≈2 at 1550 nm [12]), make KY(WO_4_)_2_ an interesting material for the realization of small, high-contrast, low-threshold, power-efficient on-chip lasers and amplifiers.

Waveguide amplifiers [10] and lasers [13,14,15] fabricated in low refractive index contrast waveguides were experimentally demonstrated in KY(WO_4_)_2_ over the past decade. Those devices were fabricated using liquid phase epitaxy (LPE) to grow an optical-quality, lattice-matched, refractive-index-engineered, doped layer onto an undoped seed crystal [16,17]. A refractive index contrast ∆n≈0.02  [16,17] could be achieved using this technique. Excellent optical performance was demonstrated in such devices, including a highly efficient thulium laser, with a maximum output power of 1.6 W and slope efficiency of ~80% [13], and devices lasing at ~1 µm, with wide tunability [14,15] and high optical gain [18].

Increasing the refractive index contrast between the core layer and the cladding increases the efficiency of the fabricated devices by increasing the field intensity on the doped material. Swift heavy ion irradiation (9 MeV carbon ions) is used to fabricate planar waveguides by introducing a buried layer with a lower refractive index approximately ~1 μm below the surface of the substrate [19]. The lower refractive index layer results from the amorphization of the crystalline KY(WO_4_)_2_ material by the energy deposited by the incident ions via electronic interactions. After an annealing step, to repair residual damage in the waveguide core, a refractive index contrast of ~0.15 was obtained with optical slab propagation losses below 1.5 dB/cm at 1550 nm [19]. A refractive index contrast ranging from 0.4 to 0.6 can be achieved by the integration of KY(WO_4_)_2_ onto a glass substrate. Sefünç et al. have demonstrated the integration of KY(WO_4_)_2_ on a glass substrate, using room-temperature curable adhesive (NOA81) and an extensive lapping and polishing procedure to thin a bulk crystal from 1 mm down to 2.4 µm [20]. Waveguides with a maximum length of 300 µm were fabricated using focused ion beam (FIB) milling. Optical losses below 1.5 dB/cm were measured for rib waveguides at 1550 nm. FIB milling of waveguides is time-consuming, and stitching errors occur during the fabrication of millimeter-long devices. Standard lithography and reactive ion etching can be used to fabricate millimeters-long waveguides in a reproducible manner. A defect-free KY(WO_4_)_2_ layer with a layer thickness between 0.90 and 1.60 µm, depending on the application, and with a maximum deviation of ±0.02 µm across the whole 10 mm × 10 mm sample surface, is required to enable this fabrication process.

In this work, we present the development of a lapping and polishing process made on thick (>300 µm) KY(WO_4_)_2_ layers. The results of applying the developed process to thin KY(WO_4_)_2_ substrates down to <2 μm thick layers are reported. Finally, the future challenges for the realization of integrated active optical devices in KY(WO_4_)_2_-on-glass are discussed.

## 2. Preparation of KY(WO_4_)_2_-on-Glass Assemblies 

The following section details the preparation of KY(WO_4_)_2_ assemblies (i.e., KY(WO_4_)_2_ bonded onto a glass substrate) for the lapping and polishing process. 

### 2.1. Three-Grade Beveled KY(WO_4_)_2_ Edges

**Figure 1:**
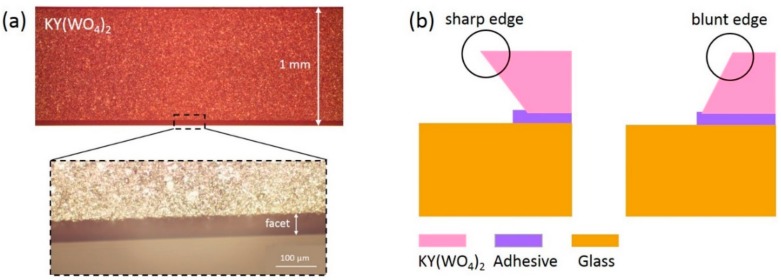
(**a**) Front view, microscope image of the edge of an as-delivered KY(WO_4_)_2_ sample from the manufacturer, showing the beveled facet and roughness. (**b**) Side-view illustration of a sharp and a blunt edge of a roughly 50 μm thick KY(WO_4_)_2_ sample. The sharp edge is vulnerable to chipping, whereas the chance of chipping is limited for the blunt edge.

The KY(WO_4_)_2_ samples used in this study (Altechna, LT) were 10 mm × 10 mm × 1 mm slabs optically polished (i.e., root mean square (RMS) roughness <1.5 nm) on one side and low grade polished (i.e., RMS roughness <30 nm) on the other. The sides of the as-delivered samples were rough, and they had beveled facets of several tens of micrometers from the polishing process (Figure 1a). The roughness on the side facets of the samples created chipped edges during the lapping process, which created defects on the sample surface. The existing beveled facets from the manufacturer were not favorable for the thinning procedure because they would have sharp, vulnerable edges if the layer was made thin, as illustrated in Figure 1b. A method is developed to create smooth three-grade beveled KY(WO_4_)_2_ facets with blunt corners (Figure 1b). The blunt corners, in combination with the different roughness, prevent the edges from chipping.

**Figure 2:**
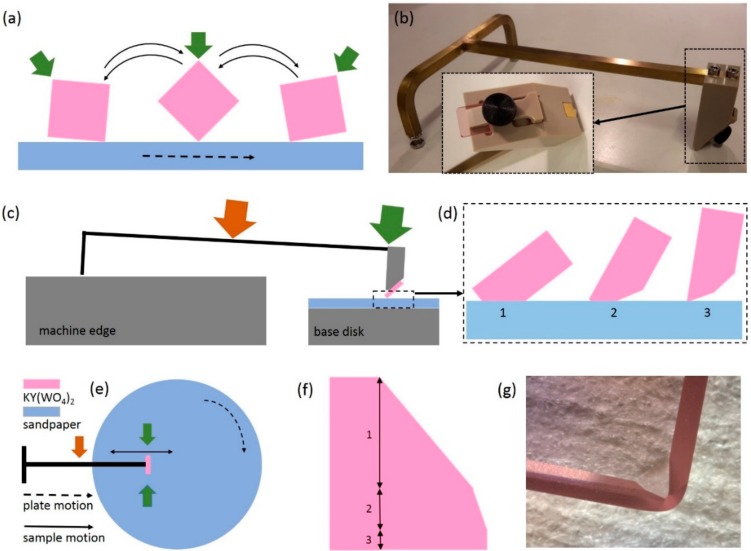
(**a**) Side view schematic representation of the motion during the process of rounding of the corners. The sample is held between the thumb and index finger at the corner pointed out by the green arrow. (**b**) Photograph of the beveling tool and a close-up (inset) view of the top of the tool with a sample (transparent pink) mounted for beveling. (**c**) Schematic representation of the relative position of the tool on the machine. The height of the base disk is increased to vary the contact angle. (**d**) Schematic representation of how to achieve different bevel angles using the setup shown in (c). (**e**) Top view schematic of the manual linear inward and outward motion of the sample on the rotating sandpaper. The head of the tool is held between the thumb and index finger to guide the sample. Pressure is exerted on the center of the bar of the tool with the index finger of the other hand (orange arrow). Note: During the process of rounding of the corners, the sample is held by hand instead of using the tool. (**f**) Schematic representation of the three areas of the beveled facets. The indicated areas, 1, 2, and 3, are consecutively made with 15 µm SiC, 3 µm Al_2_O_3_, and 0.3 µm Al_2_O_3_ sandpaper. (**g**) Close-up photograph of a beveled facets of a KY(WO_4_)_2_ sample with the mentioned grade of beveled facets, looking from the top of the crystal.

The corners of the samples were rounded, prior to beveling, to prevent chipping. This was done by manually turning the corner of the sample over wet rotating sandpaper of consecutively 15 µm SiC, 3 µm Al_2_O_3_, and 0.3 µm Al_2_O_3_ (Struers, Willich, Germany), as schematically shown in Figure 2a. The sample was held by the thumb and index finger at the corner indicated with a green arrow. After the rounding step, the beveled facets were applied to the edges of the sample. To that aim, a special tool, inspired by a traditional diamond polishing dop tool, was designed to handle the sample during the process (Figure 2b). In the tool, a sample can easily be clamped by a spring mechanism (Figure 2b). Dedicated base disks were made with a consecutively larger thickness to create the different contact angles between the sample and sandpaper, as illustrated in Figure 2c,d. The head of the tool was held between the thumb and index finger and guided the sample in an inward–outward motion over the rotating plate, onto which sandpaper was mounted (Figure 2e). At the same time, pressure was exerted on the center of the bar of the tool (orange arrow) to create the beveled facet. 

The beveled facets were made in three sections, as schematically shown in Figure 2f. Section 1, Section 2 and Section 3 were roughly 800 µm, 150 µm, and 50 µm, respectively, and were made with sandpaper of a subsequently smaller grain size (i.e., 15 µm SiC, 3 µm Al_2_O_3,_ and 0.3 µm Al_2_O_3_). The height of each segment in the facet corresponded with the targeted layer thickness that would be removed during the different lapping stages (Section 4.1). The facets should be less rough than the lapping particles used to prevent them from clustering in the edge, which can cause chipping of the edge. This can be achieved using the mentioned type of sandpaper, because fixed particles give a finer surface finish than the rolling particles used for the lapping and polishing. A photograph of the corner of one sample is shown in Figure 2g, where the different grades of beveled facets are visible.

### 2.2. Preparation of KY(WO_4_)_2_ Assemblies

**Figure 3:**
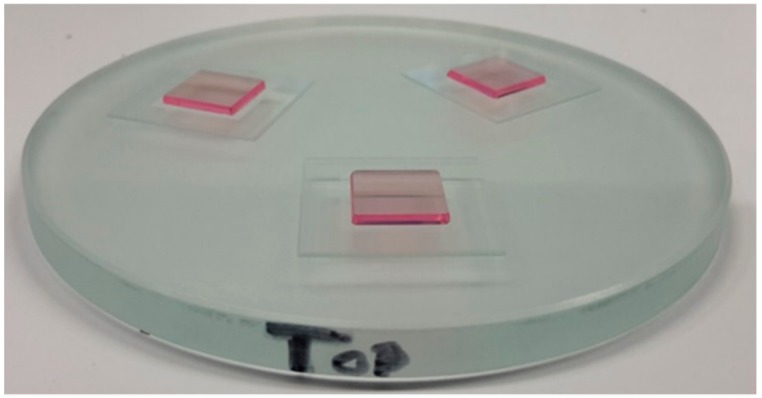
Photographs of three assemblies, each consisting of a KY(WO_4_)_2_ sample bonded with ultraviolet (UV)-curable adhesive onto a MEMpax substrate and wax mounted onto an ultra-parallel supporting glass plate for thinning and polishing.

KY(WO_4_)_2_ stacks, consisting of a KY(WO_4_)_2_ sample and a glass substrate, were assembled and eventually mounted on an ultra-parallel glass plate for the lapping and polishing process. Typically, three KY(WO_4_)_2_ assemblies are mounted on the ultra-parallel plate in a triangular configuration (Figure 3). This configuration provides a higher degree of stability for the samples on the lapping and polishing disks during the process, compared to a single mounted assembly. It also allows for more control over the load on the samples with a higher precision.

Details about the integration methods of KY(WO_4_)_2_ on glass substrates, using an ultraviolet (UV) curable adhesive, were described in our previous work [21]. The height of the individual assemblies was measured with a precision thickness measurement system (Milimar C1208, Mahr, GmbH, Göttingen, Germany), including an inductive probe (Milimar P1300, Mahr, GmbH), before mounting on an ultra-parallel plate (Ø 83 mm, thickness 6 mm, 0CON-166, Logitech, Glasgow, UK). The assemblies were used only when the deviation between the assemblies was within the measurement accuracy of the system (i.e., ±0.4 µm, including repeatability, hysteresis, and error limit of the measurement system). The assemblies were mounted in a triangular configuration on the ultra-parallel glass plate, using low-temperature melting wax (Alcowax, NikkaSeiko, Tokyo, Japan), as shown in Figure 3. For the mounting, both the assemblies and the ultra-parallel plate were heated to 65 °C on a hotplate. The assemblies were then mounted onto the given positions by melting a thin layer of wax in the required location and positioning the samples. Another ultra-parallel plate, combined with a load of 500 g, was placed on top during the cooling process to ensure an equal height of the assemblies after mounting. The height of the assemblies was checked afterward with the precision measurement system. The procedure was repeated when the assemblies deviated more than ±0.4 µm from each other.

## 3. Lapping and Polishing Equipment and Consumables

High-precision lapping and polishing equipment was required to enable the fabrication of optical high-index contrast KY(WO_4_)_2_ devices. The precision lapping and polishing machine is described in Section 3.1., followed by an explanation of the used lapping and polishing particles and disks. The “conditioning of the disk” is the preparation of the disk to obtain the required planarity and surface finish before usage for the lapping and polishing steps, which are described in Section 3.3. The general lapping and polishing process is detailed in Section 3.4.

### 3.1. Lapping and Polishing Machine

**Figure 4:**
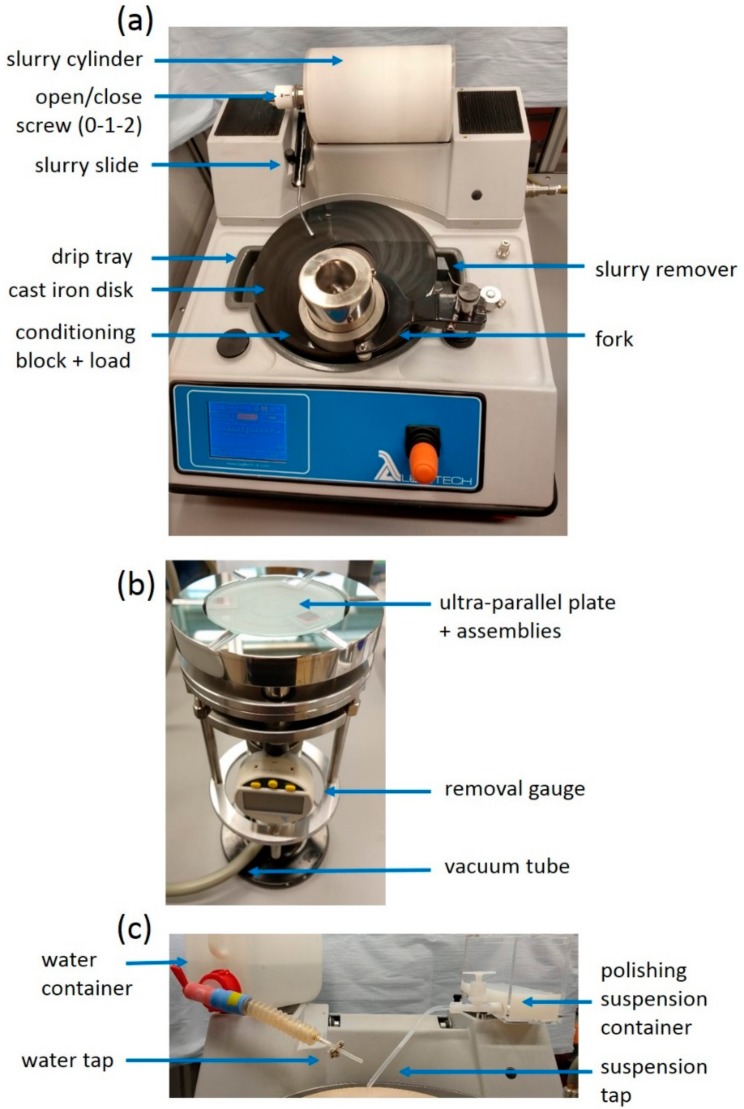
(**a**) Lapping and polishing machine (PM5 from Logitech) with conditioning block and additional load on a dedicated cast iron disk. (**b**) Precision jig holding the ultra-parallel plate with vacuum. (**c**) Close-up of water and polishing containers and tap.

The lapping and polishing process was carried out with a PM5 precision lapping and polishing machine from Logitech, shown in Figure 4a. The machine was positioned in a specially designed laminar flow cabinet, with a high-efficiency particulate air (HEPA) filter, which created an environment with a reduced number of large particles (i.e., 99.99862% of particles >0.3 µm are filtered). The polishing jig (Figure 4b) was held on the rotating disk by a positioning fork, which could be held static or swept in the desired range with the required speed. The disk rotation could be varied from 0 to 70 rpm. This range allowed precise control of both low and high removal rates. Lapping particles were mixed with deionized (DI) water in a dedicated cylinder. Prior to lapping, the lapping slurries were shaken to remove the sediment layer that typically forms at the bottom of the cylinder. The cylinder was rotated during the process to avoid sedimentation and ensure a homogeneous mixture. The slurry flow could be adjusted by opening/closing a screw (0-1-2), and the slurry was dispensed onto the disk via a slide (Figure 4a). During the polishing step, the polishing suspension was held in a container, and it was dispensed onto the disk through a tap (Figure 4c). 

The ultra-parallel plate containing the three KY(WO_4_)_2_ assemblies was held by vacuum on the precision jig (PP5, Logitech), as shown in Figure 4b. The load on the samples could be adjusted from 100 to 1500 g, and the removed-layer thickness could be monitored during the process with the digital gauge on the jig (precision of 1 µm).

### 3.2. Lapping and Polishing Materials

In this research, different types of disks were used for the lapping and polishing process of KY(WO_4_)_2_. They all had their own base disk, conditioning block, and slurry cylinder or suspension container to reduce the need for conditioning of the disk and minimize cross contamination between the different process steps.

A cast iron disk was chosen for lapping. The high hardness of the disk made it suitable to achieve a high level of planarity after the disk was properly conditioned. Calcined aluminum oxide particles with grain sizes of 9, 3, and 1 μm mixed with DI water, were used to develop a so-called coarse, medium, and fine lapping process. The aluminum oxide particles were hard (Mohs hardness 9.0) enough to allow a high removal rate on the soft and brittle KY(WO_4_)_2_ (i.e., Mohs hardness of 4.0–5.0 [22]) but not too hard to make defined scratches, which is the case for lapping of KY(WO_4_)_2_ using diamond (Mohs hardness of 10.0) particles. 

Pink (1LPE1-0600, Logitech) polyurethane disks with Shore A hardness of 94–97 were used for the development of the polishing process. This disk was selected because of its high hardness, which minimized the rounding effect on the sample surface during the long (i.e., several hours) polishing process compared to polishing on softer polishing cloths. Pre-polishing with 3 μm CeO_2_ slurry (0CON-023, Logitech, 200 g/L, Mohs hardness 8.0) was investigated prior to polishing. Those particles have a higher hardness than the 40 nm colloidal SiO_2_ suspension (OP-U, Struers Mohs hardness 7.0) selected for polishing. It is expected that the roughness from lapping will be reduced more efficiently with the pre-polishing step, due to the higher hardness of the particles, and, therefore, the final polishing time could be reduced.

### 3.3. Conditioning of Cast Iron and Polyurethane Disks 

**Figure 5:**
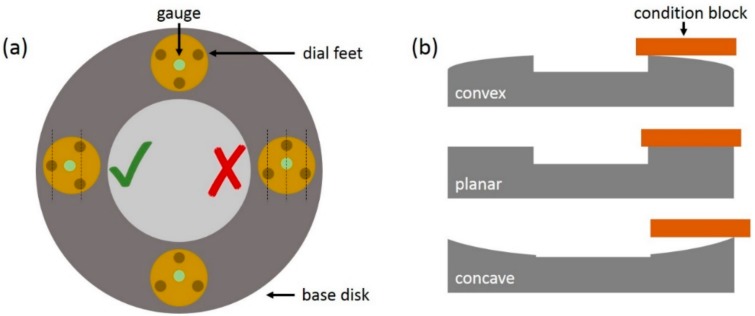
(**a**) Schematic representation of the correct and incorrect positioning of the feet of the precision measurement tool and the four measurement positions for flatness/convexity/concavity on the base disk. (**b**) Indication of the positioning of the conditioning block in the case of inside, center, and outside positioning for respectively convex-, defective-, and concave-disk conditioning.

The lapping and polishing disks needs to be checked on planarity and defects before use. The planarity can be checked with a precision meter, referenced on a granite master flat block, at four points of the base disk (i.e., position of 3, 6, 9, and 12 o’clock), as shown in Figure 5a. The precision meter needs to measure the deviation from the reference value between two points. Therefore, the legs of the precision meter have to be placed, as shown in Figure 5a, at the 9 o’clock position. An average value of the deviation is measured when the legs are placed as shown at the 3 o’clock position (Figure 5a). If the meter indicates a larger deviation than 2 µm from the zero reference, the disk is concave (i.e., positive deviation from zero) or convex (i.e., negative deviation from zero) and should be conditioned. Conditioning of the disk is also necessary if defects, such as pits or scratches, are detected after a bare-eye inspection. The conditioning of the lapping and polishing disks is carried out with a special conditioning block and an additional load of 3.5 kg. The conditioning block and load are placed at different locations on the disk, depending on the type of defect that needs to be corrected. In the case of a defect (i.e., scratch or pit), the conditioning block and weight should be placed at the center of the rim of the disk. To correct a concave disk, the conditioning block and weight should be placed toward the outer edge of the disk, and, to correct a convex disk, the conditioning block and weight need to be placed toward the inner edge (Figure 5b). It is important to ensure that the whole rim of the disk is covered by the conditioning block, to avoid the formation of a ridge.

**Figure 6:**
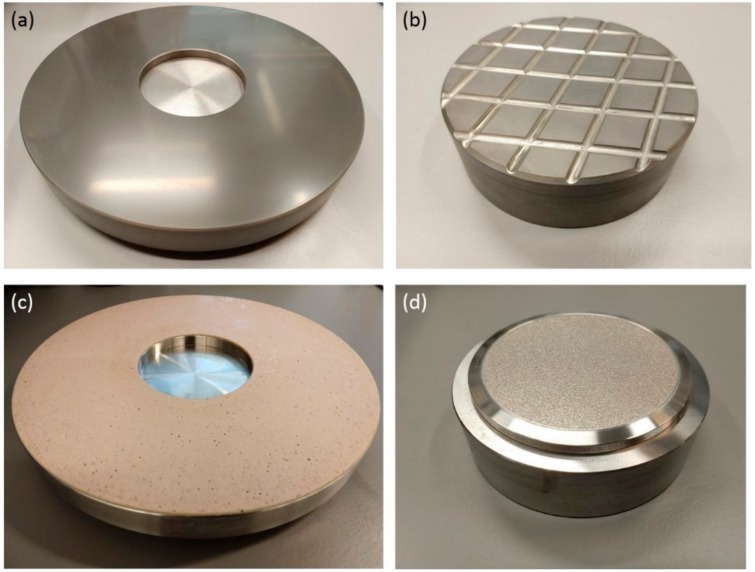
(**a**) Cast iron disk (Ø 30 cm) and (**b**) cast iron conditioning block (Ø 12.7 cm), both dedicated for medium lapping. (**c**) Polyurethane disk (Ø 30 cm) and (**d**) diamond conditioning block (200 µm diamond pellets, Ø 12.7 cm) both dedicated for polishing with 40 nm SiO_2_ suspension.

Both the lapping and polishing disk are conditioned using a dedicated conditioning block for the particular disk. The cast iron disk (Figure 6a) is conditioned using a cast iron block with grooves (Figure 6b). The grooves are wide and rounded such that they can easily be cleaned after the process. In the case of the polyurethane disk (Figure 6c), a conditioning block with 200 µm diamond pellets (Figure 6d) is used. The diamonds scratch over the surface of the polyurethane disk and remove the top surface effectively, exposing a fresh surface.

The conditioning block is positioned on the rim of the base disk at different positions depending on the curvature or defect that has to be corrected (i.e., center for defect or general condition, outer side for concave and toward the inner side for a convex-disk conditioning). Both types of conditioning blocks have to be held steady while the curvature of the disk is corrected.

A process time of 20 min with a rotation speed of 40 rpm and a load of 3.5 kg is typically sufficient to condition both the cast iron, as well as the polyurethane, disk. A calcined Al_2_O_3_ suspension with a particle size compatible to the disk (i.e., 3 µm Al_2_O_3_ slurry for the disk dedicated for medium lapping) is used with a flow rate of 10–12 drop/min during the process. Only water, with a flow rate of 1 drop/s, is used during the conditioning of the polyurethane disk.

The cast iron disk has to be cleaned with DI water and a soft tissue, and the polyurethane disk has to be cleaned with DI water and a soft nylon brush, to remove all residues after the process. Then, the planarity of the disk has to be rechecked, and the process is repeated until the specifications are met. The polyurethane disk has to be surface conditioned after planarization, to ensure an equal surface smoothness of the polyurethane layer. The diamond block is positioned at the center of the rim during surface conditioning, and it is swept across the whole surface of the rim (i.e., 0%–100% sweep) during this process.

The cast iron disks are vulnerable to rust. Therefore, they need to be stored with an oil film (e.g., paraffin liquid) to protect the disk. This oil layer can be removed with a soft tissue and isopropanol (IPA) before use. The polyurethane disk has to be cleaned thoroughly with water and a soft brush after a polishing process on KY(WO_4_)_2_ samples with polishing fluid, or else the polishing fluid crystalizes in the pores of the pad, and those crystals can cause serious scratching problems on the KY(WO_4_)_2_ samples when the disk is reused.

### 3.4. Overall Lapping and Polishing Process Description

First, the slurry was placed on a shaker to remove the sedimented layer at the bottom of the cylinder. During this time, the lapping/polishing machine was cleaned with IPA and a soft tissue to remove any residues and particles. Then, the dripping tray, base disk, jig, and other modules were cleaned and mounted on the machine. Subsequently, the base disk was checked for planarity and defects, as described in Section 3.3. Defects on the disk cause defects on the samples because of the clustering of lapping particles. Poor surface curvature of the samples is obtained when the disk is not planar because the sample is a mirror image of the surface of the disk (e.g., the sample has a convex curvature when the disk is concave). The rim of the jig should also be conditioned for an optimal grip of the jig on the base disk during the process; otherwise, the jig can make sudden movements due to the friction difference (e.g., quick rotation), which can lead to defects on either the samples or base disk. The conditioning can be done with the settings required for the particular process stage, described in Section 4.1, with the center of the jig elevated for at least 10 minutes.

The samples mounted onto the ultra-parallel plate are measured before and after each process with the precision measurement tool, in order to obtain the most accurate removal rate of material from the samples. After each measurement, the ultra-parallel plate can be mounted on the jig with vacuum, and the specified load on the samples can be set and measured with a sensitive lab scale. The process is then performed with the required settings (Section 4). A higher particle concentration than mentioned in Section 4 leads to a higher removal rate, due to the fact that the sample is in contact with a larger fraction of lapping particles. A higher load or rotation speed gives more friction between the sample surface and base disk and leads to defects due to increased stress.

The motion of the jig, flow rate of the slurry, and wetness of the disk need to be monitored carefully during the procedure. A constant flow rate is necessary to maintain the quality of the facets on the sample edges (i.e., without defects) during the process. A too-low feed rate leads to a too-dry disk, which will cause higher friction between the base disk and the sample. This eventually leads to cracks that extend through the whole layer thickness. On the other hand, a too-high feed rate will produce a wave of slurry bulging onto the samples, with the consequence of rounding and chipping of the edges. Typically, the process is interrupted, and the samples and base disk are cleaned and inspected as soon as something unexpected happens (i.e., sudden quick rotation of the jig or some unidentified particles on the base disk). All mounted components (base disk, positioning fork, dripping tray, and jig) should be cleaned thoroughly when a defect (i.e., scratch or chipped edge) occurs on the sample.

**Figure 7:**
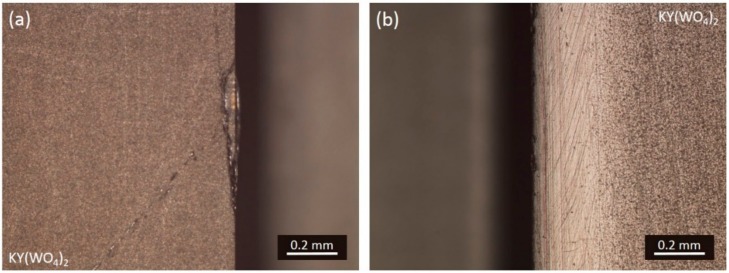
Microscope image of a fine lapped KY(WO_4_)_2_ edge (**a**) with defect and (**b**) after reparation.

A chipped edge has to be smoothened manually, consecutively with 5, 3, 1, and 0.3 µm Al_2_O_3_ sandpaper (Figure 7), and a scratch has to be removed by further lapping. An estimation has to be made on whether it is more efficient to remove a defect in a medium lapped surface with the same lapping slurry (i.e., 3 μm Al_2_O_3_ slurry) or remove it with a coarse lapping slurry (i.e., 9 μm Al_2_O_3_ slurry) before continuing the process with the medium slurry. A lapped KY(WO_4_)_2_ surface has to be defect free before proceeding to the next lapping stage (i.e., coarse → medium → fine lapping → polishing). A KY(WO_4_)_2_ layer thinner than 50 μm is too vulnerable for the reparation process because it has a high risk of cracking due to the stress in the layer. Therefore, the process has to be aborted when a defect occurs on this type of layers.

The machine and mounted components were cleaned thoroughly with DI water and IPA whenever a particular process step was completed. The ultra-parallel plate assemblies mounted on it were carefully cleaned with soap and a soft brush around the KY(WO_4_)_2_ samples. The surface of the samples needed to be cleaned with a soft tissue and IPA. It was important that no scrubbing motions were made during the cleaning of the sample, because this could have caused scratches or other defects.

## 4. Lapping and Polishing Process for KY(WO_4_)_2_

The generic lapping and polishing process described in Section 3.4 was adapted to produce optical-quality high-index contrast KY(WO_4_)_2_-on-glass devices, which require the lapping of a KY(WO_4_)_2_ sample without defects, from 1 mm down to roughly 1–2 µm. After lapping, the sample was polished to an optical grade surface (i.e., RMS surface roughness <2 nm [23]) with a high flatness (i.e., radius of curvature >200 m to achieve a maximal deviation of 40 nm across an 8 mm sample length). Both lapping and polishing processes (Section 4.1 and Section 4.2) were developed first on thick (>300 µm) KY(WO_4_)_2_ samples. The obtained process can be used to produce high-index contrast KY(WO_4_)_2_-on-glass devices.

### 4.1. Lapping Process for KY(WO_4_)_2_ Layers

The first stage, coarse lapping, was developed to enable a high material-removal rate without surface defects. The majority (~800 µm) of the sample was removed during this process step, during the final process. A slurry made from a mixture of DI water and 9 µm calcined Al_2_O_3_ particles (250.0 g/L) was made. A combination of a load on the samples of 200 g/cm^2^, a rotation speed of the disk of 35 rpm, and a slurry flow of 10–12 drop/min resulted in a removal rate of 10–15 µm/min, without defects. The effective load on the sample was reduced during this process stage due to the large thickness of material that was removed. The indicated load on the sample can be re-established midway through the process to achieve the mentioned removal rate (Table 1).

The medium lapping stage was developed to obtain a balance between a high removal rate and precise removal. For this stage, smaller lapping particles of 3 µm calcined Al_2_O_3_ mixed with DI water (208.3 g/L) were used with the same flow rate as in the previous stage. The load on the samples and rotation speed of the disk were reduced to 167 g/cm^2^ and 30 rpm, respectively. The final settings resulted in a removal rate of 5–7 µm/min.

The last stage, fine lapping, aimed to tune the layer thickness as close as possible to the 1–2 µm in the final process. A removal rate of 1–2 µm/min was achieved with 1 µm calcined Al_2_O_3_ particles mixed in DI water (187.5 g/L) in combination with a slurry flow rate of 10–12 drop/min and a reduced load on the samples and rotation speed of 133 g/cm^2^ and 25 rpm, respectively.

**Figure 8:**
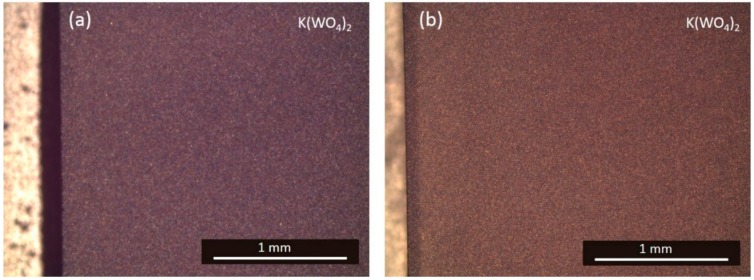
Microscope image of a KY(WO_4_)_2_ sample with (**a**) a medium and (**b**) a fine lapped surface finish.

Table 1 summarizes the settings for the lapping process. The process was carried out on a thick (i.e., >300 mm thick KY(WO_4_)_2_ layers) to verify whether this process leads to planar lapped layers with good surface quality. A microscope image of a thick sample with a smooth medium and fine lapped surface finish is shown in Figure 8a,b respectively. The flatness of this sample was measured using a profilometer (Veeco Dektak 8, Veeco, Bruker, Billerica, MA, USA), which resulted, after fitting with a quadratic function, in a surface curvature of 222 m.

### 4.2. Polishing Process for KY(WO_4_)_2_ Layers

#### 4.2.1. Polishing with SiO_2_ Suspension

A polyurethane disk and a 40 nm colloidal SiO_2_ suspension (OP-U, Struers) were used to polish thick KY(WO_4_)_2_ samples with a fine lapped surface finish. A load of 290 g/cm^2^ and a rotation speed of 25 rpm were used to create enough friction to obtain a high polishing rate. The jig was set in a sweeping motion from 30%–70% (where 50% equals the center of the rim of the base disk) to avoid the formation of a trench in the disk from the long (i.e., several hours) polishing time. The suspension was dispensed on the disk with a flow rate of 10–12 drop/min, and additional water with a flow rate of 8–10 drop/min was used to prevent the disk from drying out.

**Figure 9:**
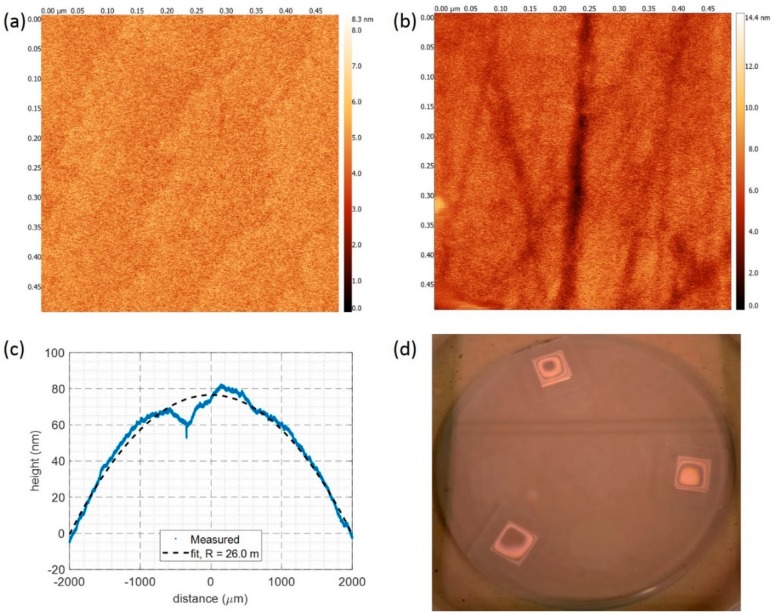
Atomic force microscope (AFM) measurements on a 0.5 µm × 0.5 µm area (**a**) KY(WO_4_)_2_ sample that was polished for two hours, with root mean square (RMS) roughness of 0.88 nm, and (**b**) an as-received sample with RMS roughness of 1.41 nm. (**c**) Measured surface profile of a KY(WO_4_)_2_ sample after two hours of polishing (blue). The measurement was carried out using a Dektak profilometer. The dashed line shows the quadratic function fit with a radius of curvature of 26.0 m. (**d**) Newton rings on the same polished samples, illuminated with a sodium lamp through an optical flat, demonstrating the high uniformity of the samples.

The surface roughness and curvature were determined for a KY(WO_4_)_2_ surface polished for 2 h (i.e., material removal rate of ~3 μm/h). The surface RMS roughness was measured with an atomic force microscope (AFM, Fast Scan, Bruker, Billerica, MA, USA) and resulted in an RMS roughness of 0.88 nm in a 0.5 µm × 0.5 µm area (Figure 9a), which was an improvement in comparison with the as-delivered sample, which possessed an RMS roughness of 1.40 nm (Figure 9b). To determine the curvature of the polished surface, a scan with a profilometer (Dektak 8, Veeco) was made, as shown in Figure 9c. A curvature of 26.0 meters was obtained after fitting with a quadratic function. This curvature was bigger than the one obtained after the fine lapping step due to the reduced hardness of the polyurethane disk with respect to the cast iron disk. However, the planarity achieved in this work after the polishing step was better than the one reported in earlier work, in which a radius of 5.9 meters was obtained for a ~1 mm thick KY(WO_4_)_2_ sample [24]. Although it used the same polishing equipment and similar process parameters used in this work, the load utilized in the referred work was much higher, namely 400 g/cm^2^. Such a high load led to a stronger pressure between the sample and the base disk, which produced more rounding, given the softness of the polyurethane disk.

Figure 9d shows Newton rings created between the sample surface and an optical flat illuminated with monochromatic light (λ = 589 nm, KEMET, Maidstone, UK). The Newton rings reveal that the surface is convex and the smaller fringes at the circumference indicate the presence of the rounding of the edges.

#### 4.2.2. Extended Polishing Time

The RMS surface roughness after 2 h of polishing was within the specifications given for an optical quality surface. However, not all features from the fine lapping stage were removed after 2 h. It could be that the load on the samples was too high, causing micro fractures from the lapping propagated further into the layer [24]. The polishing was repeated on KY(WO_4_)_2_ samples with fine lapped surface finish with the same settings as before, only with a load of 167 g/cm^2^ and a process time of 5 h. The roughness and potential defects of the fine lapped surface were still not completely removed after this polishing time. The RMS surface roughness between the defects was reduced to 0.28 nm in a 0.5 µm × 0.5 µm area.

#### 4.2.3. Pre-Polishing with CeO_2_ Suspension

**Figure 10:**
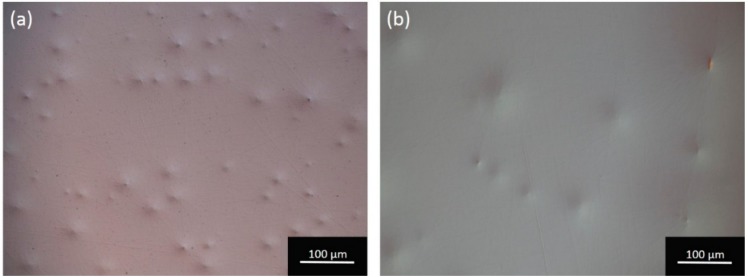
Optical microscope image in Normarski configuration of the KY(WO_4_)_2_ surface polished on a pink polyurethane disk with 3 µm CeO_2_ suspension for (**a**) 30 min and (**b**) 90 min.

A pre-polishing test with CeO_2_ particles was performed with the intention of reducing the polishing time and, therefore, also reducing the rounding of the edges. CeO_2_ particles are harder than SiO_2_ particles, so they should be able to remove the roughness from the fine lapping step more efficiently. CeO_2_ particles of 3 µm (0CON-023, Logitech, Glasgow, UK) mixed with DI water (200 g/L) were used on a pink polyurethane disk. The KY(WO_4_)_2_ samples had a fine lapped surface finish at the start of the experiment. The same process settings that was used for the fine lapping was used to create enough friction to remove the surface roughness from the fine lapping. The sweeping motion of the jig (same settings as in Section 4.2.1) was used to avoid deformation of the polyurethane disk. The surface finish after 30 min of pre-polishing is shown in Figure 10a; it reveals an orange-peel effect on the surface. The orange-peel structure could not be removed with longer polishing time (up till 90 min, Figure 10b), nor with the subsequent polishing step using 40 nm SiO_2_ suspension, with the settings specified in Section 4.2.2, for 30 min. A similar surface finish was observed after pre-polishing with a 0.5 μm CeO_2_ particle slurry (0CON-260, Logitech) on a softer polyurethane disk (ILPE1-0100, Logitech) and a polishing cloth (MD-Dac. Struers). This surface effect might occur due to a chemical reaction of CeO_2_ with the KY(WO_4_)_2_ [25,26]. Therefore, CeO_2_ was not utilized in the final process as pre-polishing suspension to achieve optically polished KY(WO_4_)_2_ layers.

Further research has to be carried out for the optimization of the polishing stage to achieve an optical quality layer with minimal surface rounding (i.e., RMS roughness <2 nm and an R >200 m).

## 5. Lapping Toward Thin Layers

The lapping stages (coarse, medium, and fine), as described in Section 4.1, were used to lap the KY(WO_4_)_2_ samples, with the intention to achieve a defect-free layer with a thickness of 1–2 μm.

Lapping of KY(WO_4_)_2_ samples bonded on flat glass substrates was tested. The samples were bonded using an optical curable adhesive and a flip-chip bonder, as described in our previous work [21].

**Figure 11:**
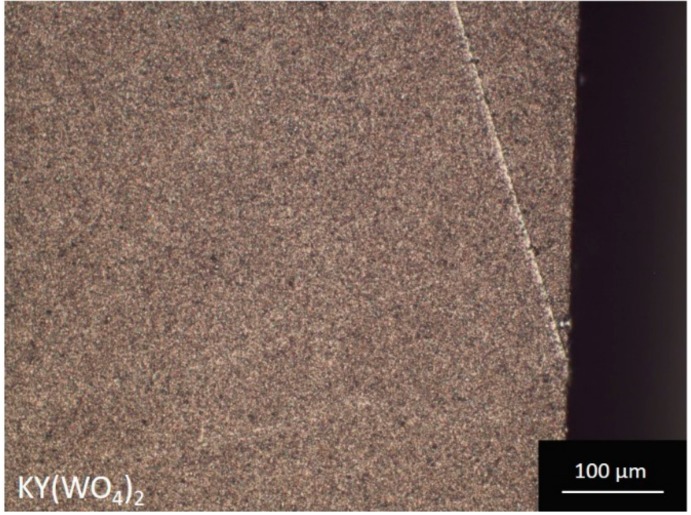
Microscope image of a chipped edge on a 56 μm thin KY(WO_4_)_2_ sample that caused a scratch on the sample surface.

Layers of roughly 800 µm were successfully removed during coarse lapping. No defects were observed on the surface or edges of the samples, and the process could be continued with medium lapping. The KY(WO_4_)_2_ layers were thinned from ~200 to ~55 µm in the medium lapping stage. Two of the three sample surfaces had at least two scratches that occurred due to chipped edges. Those chipped edges (Figure 11) were the outcome of too major resulting stress on the edges caused by the fact that the load applied on the thin samples was too large for such thin, brittle layers. The final lapping stage, which was to thin the layer to ~2 µm, could not be validated because the thin layers were never defect free after the medium lapping stage, and they were too thin to repair the defect, so further processing was aborted.

A medium and fine lapping test with lower load (load at least below 150 g/cm^2^) have to be performed on thin layers (<300 μm). In our system, a load below 100 g/cm^2^ can be reached only when at least three assemblies are mounted on the ultra-parallel plate (i.e., the most stable configuration compared to one assembly), because the spring system of the polishing jig cannot accurately maintain a weight lower than 100 g, independent of the surface area (i.e., with three assemblies, of a square centimeter each, a total load of ~110 g results in a load as low as ~35 g/cm^2^).

Another way of improving the process could be protection of the edges of the samples from chipping by, for example, using glass, which is similar to what is done during end-facet polishing of KY(WO_4_)_2_ [24]. However, this is possible only if the lapping and polishing characteristics of glass are similar to those of KY(WO_4_)_2_ using the same process. A test was performed, with the same settings as used for KY(WO_4_)_2,_ on three glass assemblies (sample and substrate) with the same dimensions as the KY(WO_4_)_2_ samples. We observed that the glass laps ~1.5 times slower than KY(WO_4_)_2_ and reaches an optical surface finish with a shorter polishing time. The different lapping and polishing characteristics of KY(WO_4_)_2_ and glass would reduce the process speed and would not add additional value to the existing process. To protect the KY(WO_4_)_2_, a material with a similar hardness (Mohs hardness 4.0–5.0) to that of the crystal, and can be cleaned with water and IPA, is needed.

## 6. Conclusions

A lapping process was developed on thick KY(WO_4_)_2_ samples (>300 µm). A surface roughness of 0.88 nm with a radius of curvature of 26.0 meters was achieved after two hours of polishing with 40 nm SiO_2_ suspension. Further work needs to be carried out to increase the planarity of the samples after the polishing step, probably by reducing the polishing time by the introduction of a pre-polishing step.

The developed lapping and polishing process was applied to the thinning of KY(WO_4_)_2_ samples from 1 mm down to a couple of micrometers. Unfortunately, scratches appeared during the medium lapping process. The scratches can contaminate the polishing material and other samples during further processing, and the samples were too thin to repair the damage. Therefore, further processing was aborted. Further process development is needed to enable the production of thin KY(WO_4_)_2_-on-glass samples in a reliable way.

## Figures and Tables

**Table 1:** Settings for the different lapping stages of KY(WO_4_)_2_ *.

Lapping Stage	Particles	Concentration (g/L)	Load (g/cm^2^)	Speed Disk (rpm)	Removal Rate (µm/min)	Logitech, UK Product nr.
Coarse	9 µm Al_2_O_3_	250.0	200	35	10–15	0CON-009
Medium	3 µm Al_2_O_3_	208.3	167	30	5–7	0CON-008
Fine	1 µm Al_2_O_3_	187.5	133	25	1–2	0CON-007

* The slurry rate was 10–12 drop/min in all stages.
